# Co-Infection Dynamics of *Helicobacter pylori* and Helminths: A Double-Edged Sword

**DOI:** 10.3390/ijms26168001

**Published:** 2025-08-19

**Authors:** Barathan Muttiah, Wathiqah Wahid, Asrul Abdul Wahab, Alfizah Hanafiah

**Affiliations:** 1 Department of Medical Microbiology and Immunology, Faculty of Medicine, Universiti Kebangsaan Malaysia, Kuala Lumpur 56000, Malaysia; barathanmuttiah@ukm.edu.my; 2 Department of Parasitology and Medical Entomology, Faculty of Medicine, Universiti Kebangsaan Malaysia, Kuala Lumpur 56000, Malaysia; wathiqah.wahid@hctm.ukm.edu.my

**Keywords:** *Helicobacter pylori*, helminth co-infection, immune modulation, Th1/Th2 balance, extracellular vesicles

## Abstract

*Helicobacter pylori* (*H. pylori*) and intestinal helminthes are common in low- and middle-income countries, where co-infection is endemic due to similar modes of transmission and poor sanitation. Whereas *H. pylori* are recognized gastric pathogens that induce gastritis, ulcers, and gastric carcinoma, helminths possess systemic immunomodulatory functions. The immunological, epidemiological, and clinical features of *H. pylori* and helminth co-infections will be discussed in this review. Key findings include that helminths induce a Th2-biased and regulatory immune response, potentially counteracting the Th1/Th17 *H. pylori*-induced inflammation and therefore limiting gastric tissue damage and cancer risk. Certain human studies and animal models concluded that co-infection would be protective against extreme gastric pathology by modulating immunity, altering gut microbiota, and by helminth-secreted extracellular vesicles. Epidemiologic data show large regional heterogeneity in co-infection prevalence with higher rates in children and rural socioeconomically disadvantaged populations. Much of the research mechanisms, however, are limited to models in the lab, and few human studies exist. Lastly, helminth co-infection is also potentially immunoprotective against *H. pylori*-associated illnesses, but greater translational research and human clinical trials are necessary. Public health policy in endemic regions must consider the complex relationships between co-infecting parasites when developing control.

## 1. Introduction

*Helicobacter pylori* (*H. pylori*) and helminth infections remain endemic in low-income settings, often coinciding due to shared risk factors such as poor sanitation and underdeveloped healthcare infrastructure [1,2,3,4]. *H. pylori* is a gastric pathogen implicated in chronic gastritis, peptic ulcer disease, and gastric cancer [5,6,7], while helminths induce chronic intestinal infections with systemic immunomodulatory effects [8,9].

**Figure 1:**
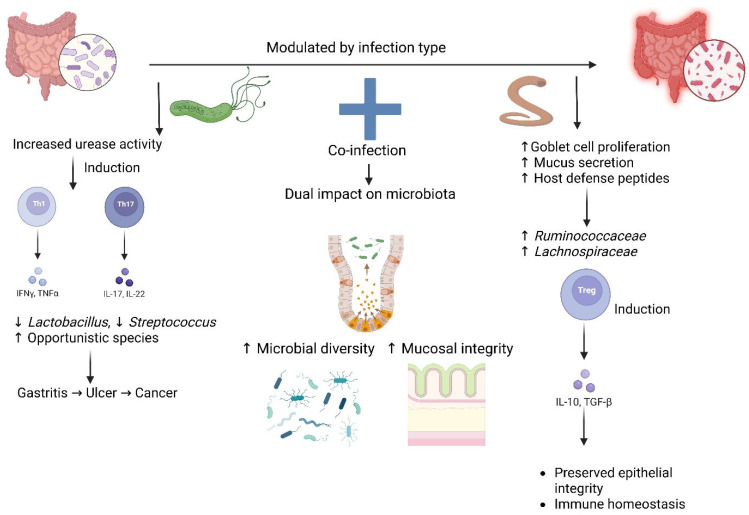
Differential impact of *H. pylori* and helminth infection on gut microbiota and mucosal immunity. Schematic representation of how *H. pylori* infection and helminth infection, individually and in co-infection, affect microbial communities and mucosal integrity. *H. pylori* increase urease activity and favor Th1 (IFN-γ, TNF-α) and Th17 (IL-17, IL-22) responses, reducing beneficial bacteria (*Lactobacillus*, *Streptococcus*) and increasing opportunistic species, leading to gastritis, ulcers, and cancer risks. Helminth infection stimulates goblet cell hyperplasia, mucus production, host defense peptide secretion, and promotes beneficial taxa (*Ruminococcaceae, Lachnospiraceae*), inducing Treg (IL-10, TGF-β) activity, ensuring epithelial integrity and immune homeostasis. Co-infection can optimize microbial diversity and maintain mucosal barrier function, unlocking a potential modulatory function in disease progression.

Interestingly, *H. pylori* elicit a robust pro-inflammatory Th1 and Th17 immune response, which is accountable for gastric tissue damage [10]. By contrast, helminths tend to skew the host immune system towards Th2 and regulatory responses, which can dampen *H. pylori*-induced inflammation [11]. Helminths such as *Ascaris lumbricoides*, *Trichuris trichiura*, and *Schistosoma* spp. induce a strong Th2 and regulatory T cell (Treg) response, characterized by the release of IL-4, IL-5, IL-13, and IL-10 [12]. This has a tendency to suppress the Th1-dominant, pro-inflammatory response typically induced by *H. pylori*, which might suppress gastric inflammation but stimulate bacterial persistence [9]. In contrast, helminths such as *Strongyloides stercoralis* and hookworms could initiate mixed Th1/Th2 responses and modulate *H. pylori*-related pathology in a more variable fashion. Immune modulation is species-specific and could underlie geographic variation in the clinical presentation of *H. pylori* infection [13]. These helminths have the ability to modulate immunity, and this impacts *H. pylori* pathogenesis and disease progression [8,13]. This mini review recapitulates the immunological interactions, epidemiology, and clinical relevance of *H. pylori* and helminth co-infections, with a focus on highlighting important findings from animal models and human populations. Figure 1 shows the overall potential co-infection dynamics of *H. pylori* and helminths.

## 2. Immunological Interactions in Co-Infection

### 2.1. Immune Response to H. pylori

*H. pylori* infection elicits a severe and chronic immune response following gastric mucosal colonization. The reaction is Th1-skewed, characterized by the secretion of pro-inflammatory cytokines IL-2, IL-12, TNF-α, and IFN-γ [14]. These cytokines not only activate macrophages and cytotoxic T cells but also maintain a pro-inflammatory microenvironment that drives chronic gastritis. Secondly, *H. pylori* elicit a Th17 response, characterized by cytokine secretion of IL-17A, IL-17F, IL-21, and IL-22. The IL-17 cytokine family members are most important in recruiting and activating neutrophils and the augmented generation of reactive oxygen species (ROS), nitric oxide, and proteolytic enzymes [15]. Immune mediators are accountable for epithelial barrier disruption and extended mucosal damage. Neutrophils and mast cells, attracted to the infected zone, discharge granules with proteases and inflammatory mediators that add to tissue damage [16]. Mast cells can also modulate epithelial cell function and induce angiogenesis, playing roles in both inflammation and the precancerous changes in chronic *H. pylori* infection [17]. Despite this potent immune stimulation, *H. pylori* will often persist for decades, suggesting that the organism has acquired sophisticated immune evasion mechanisms. These include the modulation of T cell responses, interference with dendritic cell development, and induction of suppressive Tregs that abate inflammatory responses [18]. Critical virulence factors such as CagA and VacA play critical functions whereby CagA induces Treg differentiation via STAT3 signaling and promotes PD-L1 expression on gastric epithelial cells, thereby inhibiting CD8^+^ T cell cytotoxicity, whereas VacA induces mitochondrial damage to T cells, suppresses Th1 cytokine production (IFN-γ), and disturbs the homeostasis of Th1/Th2 towards tolerance [18,19]. The outer membrane protein antigenic drift (BabA, SabA) and molecular mimicry via Lewis antigen-like O-antigens in lipopolysaccharide (LPS) reduces pattern recognition receptors and adaptive immune recognition [20]. Lipid A modification in LPS also suppresses antimicrobial peptides and innate immune receptor activation [15]. *H. pylori* also inhibit innate immune recognition by altering surface ligands to avoid Toll-like receptor and C-type lectin receptor activation and by interfering with antigen presentation in dendritic cells [21]. These mechanisms create an “immune suppression–chronic activation” cycle, where inflammation is perpetuated but clearance is negated [5]. In immunosuppressed hosts, compromised immune surveillance increases the effectiveness of such avoidance strategies, allowing the bacterium to gain long-term colonization at the cost of chronic gastritis, mucosal injury, and increased susceptibility to ulceration and carcinogenesis [5,6,7].

### 2.2. Immune Response to Helminths

The host immune response to helminth infections is typically most commonly a type 2 immune profile, which involves a synergistic effect between innate and adaptive immune components that collectively aim to kill the parasite, induce tissue repair, and avoid excessive inflammation [22]. In helminth infection and migration through mucosal surfaces such as the gastrointestinal tract, lungs, or skin, the tissue damage that ensues serves as an initiating stimulus, and the epithelial cells release alarmins, crucial cytokines such as IL-25, IL-33, and thymic stromal lymphopoietin (TSLP) [23]. These alarmins activate type 2 innate lymphoid cells (ILC2s), dendritic cells (DCs), mast cells, eosinophils, and basophils to generate signature type 2 cytokines including IL-4, IL-5, IL-9, and IL-13. These cytokines drive naïve CD4+ T cells towards T helper 2 (Th2) cells, which is a hallmark of helminth infection [24]. Th2 cells also continue to maintain type 2 immunity through the promotion of the production of IL-4, IL-5, and IL-13, which are required for B cell isotype class switching to IgE and IgG4, eosinophil activation, and alternatively activated macrophage (M2 macrophage) stimulation involved in tissue remodeling and parasite destruction [25,26]. Eosinophils and mast cells release cytotoxic granules such as major basic protein and eosinophil peroxidase, which are capable of mediating ADCC to kill or damage helminths [27]. Goblet cell hyperplasia and the hypersecretion of mucus trap parasites and enable them to be expelled through coordinated smooth muscle contraction. In chronic helminth infections, there exists a strong regulatory immune element with the proliferation of Tregs and release of anti-inflammatory cytokines such as IL-10 and TGF-β [28]. This immunoregulatory environment controls the over-activation of inflammation and thus preserves tissue pathology but allows parasites to live in harmony in a usually asymptomatic state [29]. The immune reaction is tissue-specific as well, especially in muscle tissue, eosinophils may discharge IL-10 to induce tolerance towards encapsulated larvae during the chronic infection stages. Overall, helminth-induced immunity is a prime example of an exquisite balance between immune regulation and parasite clearance, enabling the survival of the host with minimal tissue damage while permitting the parasite to survive [30]. In addition to the above characteristic cell- and cytokine-mediated pathways, helminths also release extracellular vesicles (EVs) with proteins, lipids, and regulatory RNAs that can influence innate and adaptive immunity [31]. The EVs have been shown in single infection models to induce anti-inflammatory cytokine production (IL-10, TGF-β), augment regulatory T cells, and modulate the antigen-presenting cell function [32]. Elucidation of helminth EVs has not yet been conducted in *pylori*. Since helminth co-infection with *H. pylori* co-infection is not addressed, it is reasonable to propose that such vesicles might direct the host response toward a Th2 or regulatory phenotype, perhaps suppressing the pro-inflammatory setting normally seen in *H. pylori* infection [33]. This speculation needs to be explored in models of co-infection to further characterize the role of helminth-derived EVs in modulating gastric immune responses.

### 2.3. Modulatory Effects of Helminths on H. pylori

Emerging evidence suggests that helminth co-infection may modulate the immune response to *H. pylori*, potentially blunting the underlying gastric pathology of the bacterium through immune polarization, regulatory controls, and the preservation of gastric homeostasis; however, these observations remain preliminary and require further confirmation [13,34]. *H. pylori* tend to elicit a vigorous Th1- and Th17-polarized response, which is characterized by the increased secretion of pro-inflammatory cytokines including IL-2, IL-12, IL-18, TNF-α, IFN-γ, IL-17A, IL-17F, IL-21, and IL-22, the collective action of which contributes to chronic gastric inflammation, recruitment of neutrophils, mast cell activation, and continuous tissue damage in the form of gastric atrophy, epithelial dysplasia, and in certain cases, gastric cancer [35]. By contrast, helminth infection elicits a dominant Th2-type response, which allows the release of IL-4, IL-5, IL-10, and IL-13, cytokines that counteract the Th1 response and promote anti-inflammatory and tissue repair processes. These cytokines inhibit gastric inflammation through the inhibition of pro-inflammatory mediators and a boost to the activity of Tregs, particularly FoxP3+ Tregs, which also augment immune tolerance and prevention against mucosal damage [36]. Animal models, especially transgenic INS-GAS mice co-infected with *H. pylori* and helminths, consistently show reduced gastric atrophy and dysplasia despite chronic bacterial colonization, a process that is partially attributed to the modulation of cytokine patterns and a healthy gastric microbiota [37,38]. Similarly, in human populations from helminth-endemic regions such as Venezuela and Colombia, co-infection has been associated with lower pro-inflammatory cytokine production and an increased IL-4/IFN-γ ratio, suggesting an immunoprotective milieu [39]. Such immunological processes can be held accountable for epidemiological observations such as the “African enigma,” in which a high prevalence of *H. pylori* infection is not accompanied by a high incidence of gastric cancer, possibly due to the immunomodulatory effects of helminths [40,41,42,43]. Their protective role extends outside of the stomach, and it has been discovered that exposure to helminths can inhibit or delay the onset of Th1-mediated autoimmune diseases and inflammatory conditions like multiple sclerosis and Crohn’s disease [44]. Miraculously, helminths can even modulate systemic immune cell migration, as shown in schistosome-*H. pylori* co-infection models where antigen-experienced CXCR3+ T cells are repelled from the stomach, suppressing gastric inflammation while simultaneously reducing liver fibrosis [45,46]. Epidemiological data also confirm the frequent co-existence of these infections in environments of unhygienic living, with rates of co-infection from 1% to over 70%, particularly in Latin America, Africa, and in some areas of Asia [47]. For instance, studies conducted in Mexican, Chinese, Egyptian, Iranian, Ugandan, Colombian, and Venezuelan populations have reported rates of adult and children co-infection as 50–74% and 30–63%, respectively [48,49]. The incidence of *H. pylori* and helminths generally overlaps since they share identical transmission routes, and co-infection strongly correlates with polyparasitism and greater virulence *H. pylori* strains like cagA [49]. Concurrent infection with *Opisthorchis viverrini* and *H. pylori* in Thailand aggravated biliary fibrosis and highlighted the possible synergistic impact of parasite–bacteria interactions on the severity of the disease [50]. However, not all interactions are harmful whereby certain helminths have been reported to suppress gastric carcinogenesis by *H. pylori* [51]. Seroprevalence research in Colombia reported an increased prevalence of *Ascaris lumbricoides* in low gastric cancer areas, indicating a protective association [52]. In spite of this increasing evidence base, there are still important gaps in the immunological interactions of these infections in humans. Much of the mechanistic information has come from animal models, and very few clinical trials have directly studied these interactions. This is particularly observed among indigenous peoples such as Malaysia’s Orang Asli, where co-infection data are scarce despite a high endemicity of both infections. Hence, translational research in the future must be directed toward clinical research on how helminth co-infections alter the immunopathogenesis of *H. pylori* and of their therapeutic modulation potential for the prevention of severe gastric outcomes [53]. Table 1 summarizes the distinct immune pathways activated by *H. pylori* and helminth infections individually and highlights how co-infection alters immune dynamics.

## 3. Epidemiology of *H. pylori* and Helminth Co-Infection

Globally, the global prevalence of *H. pylori* co-infection with helminths or other intestinal parasites varies widely by geographic location, and the combined prevalence in symptomatic groups has been reported to be approximately 31% (95% CI: 18.66–43.39%), though this conceals significant heterogeneity (I^2^ = 98.9%) that is explained by geographic, demographic, and methodological factors [54,55]. The epidemiology is distinct in terms of geographic and demographic distribution, with the disease being highly prevalent and focal in low- and middle-income nations (LMICs) where poor hygiene conditions, low access to clean water, and high parasite loads are the norm [55]. Studies mainly from Africa, Asia, and Latin America document these co-infections more extensively; by contrast, there is limited data from high-income countries where helminth infections are less endemic. Egypt stands at the highest reported co-infection rates in Africa at around 39.8% (95% CI: 27.8–51.9%), largely attributed to extensive cases of both *H. pylori* (~69.4%) and intestinal parasites (~51.4%), [55] whereas Ethiopia has some of the lowest estimates at approximately 5.9% (95% CI: 4.1–7.6%) [56]. Uganda has intermediate rates, with about 30.2% of children co-infected, whereas Nigeria’s reported rates are much lower (~7%), despite the prevalence of *H. pylori* infection being as high as 89.7%, suggesting widespread transmission and early acquisition [57,58]. Sudan has wide variation with a pooled prevalence of approximately 29.3%. In these environments, methods of diagnosis significantly influence prevalence rates, such that the methods that incorporate microscopy and molecular PCR detection have higher rates (~48.7%) than microscopy alone (~28.1%) [59]. In Latin America, Colombia, Tumaco, and Pasto it is extremely endemic, with *H. pylori* infections at ~93% and helminth infections at 25–54%, thus providing co-infection rates of 21–45% in children, especially in low gastric cancer incidence areas [60]. Mexico also adds considerable burdens, with 50–70% of *H. pylori*-infected children being co-infected with one or more intestinal parasites [57,61]. In Iran, ~54% of children with recurrent abdominal pain are H. pylori-positive, with 10–30% co-infected with protozoans or helminths [62]. Turkey also reports 15–45% co-infection rates in symptomatic children, primarily with Giardia lamblia [63]. In Yemen, the incidence of *H. pylori* infection is around 30% among dyspeptic patients who are frequently co-infected with protozoa such as *Entamoeba histolytica* and *Giardia lamblia*, although helminths are less frequently found [64]. In Thailand, co-infection with *H. pylori* was shown to contribute to the pathogenesis of chronic opisthorchiasis and the progression of periductal fibrosis of bile, while the fish tapeworm is the only parasite associated with cancer, such as cholangiocarcinoma, and other parasites are rarely related to malignancy [65]. Interestingly, concurrent coexistence with helminths has been associated with low parasite-induced cancer occurrence, as seen in Colombia, where more frequent seropositive *Ascaris lumbricoides* was observed among subjects in low gastric cancer regions compared to subjects from high-incidence regions [52,66]. In every region, children tend to have a greater prevalence of co-infection compared to adults, which supports early exposure in high-endemic settings, because several behavioral, biological, and environmental factors intersect to heighten the infection likelihood. Children have a higher likelihood of engaging in outdoor play, often barefoot or with skin contact with the ground, which increases exposure to infective stages of *Ascaris lumbricoides*, *Trichuris trichiura*, and hookworms [67,68]. Hand-to-mouth behavior, in addition to unhygienic practices and unavailability of clean water or sanitation facilities, also increases transmission [69]. Biologically, their developing immune systems have a lower efficiency in developing protective defenses, and malnutrition weakens immunity such that infections are more likely and harder to clear [70]. Low parental education, poor housing conditions, and limited healthcare resources, which are factors of socioeconomics, increase exposure risk and reduce prevention opportunities [71]. Aside from immediate health effects, including anemia, malnutrition, and growth retardation, long-term helminthic infection in childhood can affect cognitive development, lower achievement in school, and sustain poverty cycles [72]. These trends underscore the necessity for evidence-based integrated interventions that incorporate mass deworming, improved water and sanitation structures, nutritional interventions, and community health education to break the transmission–disease cycle. Overall, the greatest rates and greatest variability are found in resource-poor settings such as some parts of Africa, but high burdens are also present in Latin America and parts of Asian countries, pointing out that local epidemiology, socio-economic determinants, and diagnostic methods importantly shape the patterns realized in *H. pylori*–helminth co-infection. This demographic skew may be of major clinical importance, as co-infection has been observed to influence the course and severity of *H. pylori*-associated gastric disease. Table 2 shows the *H. pylori*–helminth/intestinal parasite co-infection prevalence by region/country.

## 4. The Socioeconomic Factors That Influence the Geographic Distribution of These Co-Infections

Geographical and socioeconomic differences play a dominant role in shaping the epidemiology, transmission, and clinical presentation of *H. pylori* as well as helminthic infections. For instance, *H. pylori* infection rates can reach over 70% in certain African nations, whereas rates remain much lower in developed parts of the world like North America and Oceania, where rates of *H. pylori* infection are frequently below 40% and 25%, respectively [73]. Likewise, infections of helminths spread through soil, contaminated water, or raw meat are strongly prevalent in the very same disadvantaged settings, underlining an association of co-endemicity [74]. In the vast majority of developing countries, *H. pylori* are acquired in early childhood in most cases, typically prior to five years, and are permanent if left untreated during their lifetime, reflecting poor public health control activities and low awareness. Conversely, individuals in developed nations experience gradual acquisition with growing age due to better hygiene, socioeconomic status, and health education [75]. Local variations within countries whereby urban versus rural residences, for example, can also influence prevalence, with rural areas typically having higher infection rates due to inferior infrastructure and healthcare [76]. Socioeconomic status (SES) is the strongest determinant of both *H. pylori* and helminth infection. Low SES, with inferior living conditions, crowding, lower parental education, and inadequate health literacy, facilitates fecal–oral modes of transmission common to these diseases [77]. Furthermore, certain social determinants such as occupational class, marital practices, and even prolonged working hours have been associated with vulnerability to infection and treatment compliance. Education level plays a double role: not only does it influence the risk of infection, but it also influences health-seeking behavior and the likelihood of eradication therapy success [78]. Failure in treatment has been reported to occur more among females and rural residents, perhaps due to variations in antibiotic resistance, utilization of healthcare services, or medication compliance [79]. These socioeconomic conditions foster “syndemics,” in which biologically interacting infections co-occur and amplify one another’s adverse health effects. Lower education levels result in bad hygiene practices, low health literacy, and the late seeking of treatment, which result in both an increased susceptibility to infection and failure of treatment [80]. Women and rural populations have been reported to experience increased treatment failure rates, perhaps because of gender-related disparities in access to care and antibiotic resistance patterns [81]. In addition, the endemically poor coverage of co-infections among impoverished populations, in contrast to global gains in health, speaks of an “intergenerational poverty trap” driven by chronic deprivation and inadequate public health interventions [82]. This is compounded by the “birth cohort effect,” where children born into poor environments are at a sustained risk throughout their life as a result of low socioeconomic mobility. Slums in cities and rural settlements continue to be hotspots for transmission due to poor housing conditions and environmental exposure [83]. Even in wealthier nations, socioeconomically disadvantaged populations have excessively high infection rates, which suggests entrenched inequalities [84]. Overall, a socioeconomic disadvantage heightens the exposure and susceptibility to infection with *H. pylori* and helminths and maintains their transmission from one generation to the next. Control of the structural determinants through improved water and sanitation, housing, education, and access to equitable healthcare is imperative in breaking the cycle of co-infection and reducing the worldwide burden of these illnesses [85].

## 5. Microbiota Modulation During *H. pylori* and Helminth Co-Infection

The human gut microbiota is a highly complex and dynamic ecosystem that responds sensitively to infectious and immunologic stimuli, particularly in the case of *H. pylori* and helminth co-infection, which induces dramatic alterations in microbial composition and function through both direct colonization effects and indirect immune-mediated mechanisms [86]. Despite *H. pylori* primarily infecting the stomach, it also induces systemic effects along the entire gastrointestinal tract; that is, a significant reduction in gastric microbial α-diversity and dysbiosis caused by suppression of the commensal taxa like *Lactobacillus* and *Streptococcus* and an increase in opportunistic or pro-inflammatory species [87]. This effect is partially driven by *H. pylori’s* urease activity, which elevates gastric pH, thus promoting the viability of otherwise acid-sensitive microorganisms, and secondarily, it influences intestinal microbial populations by altering bile acid compositions, nutrient assimilation, and host immune signal changes that alter the equilibrium among overbearing bacterial phyla such as Firmicutes, Bacteroidetes, and Proteobacteria [88]. Such perturbations have been associated with systemic metabolic derangements such as obesity and insulin resistance. Alternatively, infection with helminths is more likely to yield a compositional shift within the microbiota, with rises within certain bacterial families of groups like Ruminococcaceae and Lachnospiraceae, both large SCFA-producing members, including butyrate, acetate, and propionate [89,90,91]. These SCFAs play significant roles in maintaining gut epithelial integrity, modulating immune responses, and providing energy to colonocytes [92]. However, the specific microbial changes vary with the helminth species and host-specific factors like genetics, diet, and environmental exposures, resulting in the heterogeneity of human studies [93]. Meanwhile, the anti-inflammatory environment regulates gut epithelial physiology through the increased goblet cell proliferation, secretion of mucus, and host defense peptides, all of which restructure the gut microbial ecosystem. As a counterpoint, the microbiota reacts by producing SCFAs, which in turn enhance Treg differentiation and maintenance of immune tolerance, establishing a tripartite feedback loop among host immunity, helminths, and resident microbiota [94]. In addition, the helminth–microbiota interaction is not restricted to immune modulation but also affects host metabolism and epigenetics [95].

The co-infection of *H. pylori* and intestinal helminths has a dual and intricate impact on gut microbiota, modulating both microbial diversity and composition, and, concurrently, remodeling host immune responses [96,97]. Helminth co-infection appears to improve some of the adverse microbiota changes associated with *H. pylori* [98]. In mouse models, helminth co-infection has been shown to decrease *H. pylori*-induced gastric atrophy and epithelial dysplasia and preserve the stomach’s colonization resistance to the lower gastrointestinal microbiota [13]. These findings suggest that helminths can aid in maintaining microbial compartmentalization and prevent colonic flora translocation into the gastric niche, a mechanism involved in gastric carcinogenesis [99]. In addition, helminths can modulate the gut microbiota by metabolic and epigenetic pathways, specifically through the production of SCFAs as signaling molecules and histone deacetylase inhibitors, which modify the gene expression involved in immune tolerance and gut barrier function [99,100]. There is little known information to date, however, as most mechanistic data have been derived from murine models that do not precisely mirror human gastrointestinal physiology, and human research is often confounded by food heterogeneity, antibiotic use, and environmental confounders. Thus, in-depth metagenomic and metabolomic analyses of co-infected populations across various geographic and socioeconomic contexts are required to identify particular microbial signatures and functional pathways involved. In general, helminth co-infection has the potential to abrogate *H. pylori*-related microbial and immunologic abnormalities, offering protective advantages through increased microbial diversity, immune homeostasis, and the preservation of mucosal integrity, that individually might slow the evolution of premalignant gastric lesions and reduce the incidence of *H. pylori*-associated disease, particularly in high-prevalence, low-resource settings. That said, not all helminth-induced changes in microbiota are therapeutic whereby some, such as increased Bacteroidetes and decreased Firmicutes, can exacerbate other pathologies including bacterial colitis [101]. The net effect of co-infection on gut microbiota is therefore context-dependent, being modulated by factors from the species of helminth to host genetics and environmental exposures [102]. Collectively, these findings underscore the multifaceted interactions among *H. pylori*, helminths, and the intestinal microbiota and suggest that helminth-mediated microbial modulation can exert a buffering effect on *H. pylori*-induced inflammation and disease. Clarification of such dynamics opens the door to microbiome-targeted therapies including probiotic administration or helminth-based immunomodulators as adjunctive measures in *H. pylori* disease prevention and treatment [103].

## 6. Discussion

The complex immunological relationship between helminth co-infections and *H. pylori* is illuminated by this review, with an illustration of how Th2 and regulatory immune responses evoked by helminths could down-modulate the *H. pylori*-induced Th1/Th17-mediated gastric inflammation [104]. Such immune modulation provides a plausible account for epidemiological patterns such as the “African enigma,” where the endemicity of *H. pylori* is not correlated with an increased incidence of gastric cancer. Immunosuppressive properties of helminth-evoked cytokines (IL-4, IL-10, and TGF-β) and Tregs and their role in influencing microbiota balance and mucosal homeostasis increases the likelihood that helminths can modulate *H. pylori*-associated pathology, including precancerous lesions [105]. However, some significant gaps of knowledge exist. Most mechanistic findings are from murine models, which may not fully reflect human immune responses or microbiota diversity, and large-scale human cohort data from endemic regions are limited. The proposed function of helminth-derived extracellular vesicles in modulating *H. pylori*-induced inflammation is speculative at this time and requires further molecular characterization and in vivo validation [106].

It must be pointed out that these immunologic results are not proof against mass deworming. Infection with helminths remains a major cause of morbidity, loss of physical and cognitive capacity in children, and vulnerability to other infections due to their immunomodulatory effects [107]. Mass deworming is, and will remain, an important public health intervention in affected regions. In fact, deworming initiatives need to be tailored to local infection patterns because the prevalence, intensity, and age distribution of helminth infections vary geographically. Along with this, a community-wide mass drug administration (MDA) may be required in high-transmission environments or where adults are a significant source of infection, while school-based treatment initiatives are sufficient where children carry most infections [108]. Local ecological environments, water, sanitation, and hygiene (WASH) infrastructure, previous treatment coverage, and socio-behavioral determinants also affect reinfection risk and program success [109]. Accounting for these determinants renders interventions effective, sustainable, and in concordance with transmission dynamics, as demonstrated by multi-country trials like the DeWorm3 trial [110].

The overall findings presented here instead highlight the biological complexity of helminth–*H. pylori* co-infection, which may have implications both for gastric disease pathogenesis and for the planning of clinically applicable studies in context.

**Figure 2:**
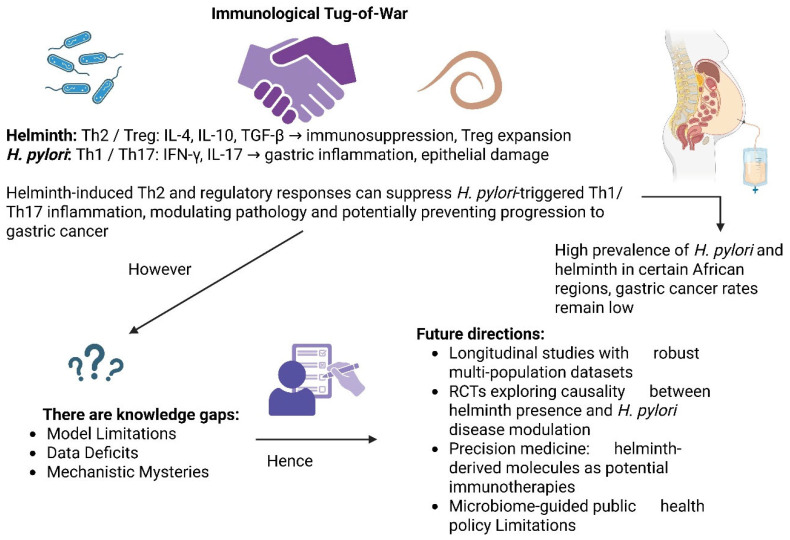
Immunological interaction of co-infection of helminths and *H. pylori* infection. Schematic representation of the “immunological tug-of-war” between helminth-induced type 2/regulatory immune responses (Th2, Treg; IL-4, IL-10, and TGF-β) and *H. pylori*-induced type 1/type 17 inflammatory responses (Th1, Th17; IFN-γ, IL-17). Helminth-mediated immune modulation can potentially dampen *H. pylori*-induced gastric inflammation and lower the risk of development of gastric cancer, as speculated in the backdrop of the “African enigma.” Knowledge gaps include model limitations, data shortages, and limited mechanistic understanding. Future research fronts involve longitudinal multi-population cohort studies, randomized controlled trials, exploration of helminth-derived molecules as potential immunotherapies, and implementation of microbiome-informed public health interventions.

Cautious interpretation of these findings is required, recognizing the limitations in the currently available body of evidence, including small sample sizes, diagnostic test heterogeneity, and geographic variations in infection rates and host genotypes [111]. Furthermore, while helminthic immunomodulation appears beneficial under some experiment conditions, it may indirectly result in suboptimal outcomes such as a loss of vaccine efficacy or enhanced susceptibility to other pathogens. Future longitudinal cohort studies and randomized controlled trials should analyze the effect of co-infection with helminths on the outcome of *H. pylori* disease across different populations [112]. At the same time, studies investigating helminth–microbiota–host interactions using metagenomics and metabolomics might uncover therapeutic avenues, such as helminth immunotherapy or microbiome interventions. To solve the co-infection complexity is not only crucial to an enhanced clinical understanding, but also for the formation of more nuanced and fair public health policy [113]. Figure 2 illustrates the immunological interaction between helminth infections and *H. pylori* in co-infections.

## 7. Conclusions

The dynamic interactions between *H. pylori* and helminth co-infections represent a crucial but understudied field of infectious disease immunology of specific interest to low- and middle-income countries where both pathogens are highly endemic. This review has illustrated how helminths, through their immunomodulatory effects principally by enhancing Th2 and regulatory immune responses, can mitigate the pro-inflammatory damage caused by *H. pylori*, potentially reducing gastric disease complication risks, including cancer. These protective interactions not only explain observed epidemiological heterogeneity but also challenge existing paradigms for deworming and gastric disease control. Even with these findings, the current evidence base is largely founded on animal experimentation and observational studies, and there is an urgent need for well-designed translational and clinical studies to confirm these mechanisms in human populations. Moreover, public health programs within endemic regions must consider the broader implications of co-infections and their immunological impact when implementing strategies like mass deworming or *H. pylori* eradication programs. The integration of co-infection biology knowledge into health policy can improve disease prevention and therapeutic outcomes in resource-limited settings.

## Figures and Tables

**Table 1:** Summary of immunological responses and modulatory effects during *Helicobacter pylori* and helminth co-infection (↑: upregulation, ↓: downregulation and →: leading to).

Aspect	*H. pylori* Infection	Helminth Infection	Co-Infection Effects
Immune Response Type	Th1 and Th17-dominant	Th2 and Regulatory (Treg)	Immune modulation: Th1 suppression, increased Treg/Th2 balance
Key Cytokines Involved	IL-2, IL-12, TNF-α, and IFN-γ (Th1); IL-17A, IL-17F, IL-21, and IL-22 (Th17)	IL-4, IL-5, IL-9, IL-13, IL-10, and TGF-β	↑ IL-4, IL-10, ↓ IFN-γ, ↓ IL-17A → anti-inflammatory environment
T Cell Polarization	Th1/Th17 skewing	Th2 skewing, Treg induction	Shift from Th1/Th17 to Th2/Treg profile
Innate Immune Cells Activated	Neutrophils, mast cells, and macrophages	Eosinophils, mast cells, basophils, and ILC2s	Balanced activation, reduced pro-inflammatory damage
Tissue Effect	Chronic gastritis, epithelial damage, gastric atrophy, and potential cancer	Tissue remodeling, parasite clearance, and mucosal repair	Reduced gastric inflammation, preserved mucosal integrity
Microbial Impact	Gastric dysbiosis, decreased α-diversity	↑ SCFA-producing bacteria (*Ruminococcaceae*)	Improved microbial diversity, restoration of homeostasis
Immunoregulation	Limited via H. pylori immune evasion (Treg induction)	Strong: IL-10, TGF-β, and Treg proliferation	Enhanced Treg-mediated suppression of gastric pathology
Clinical Outcomes	Risk of gastric ulceration, metaplasia, and adenocarcinoma	Usually asymptomatic, chronic infection	Potential protection against *H. pylori*-associated disease progression
Experimental Evidence	Well-established in animal and human models	Strong in animal models, observed in endemic populations	Animal studies (INS-GAS mice); observational studies (Colombia, Venezuela)
Gaps in Knowledge	Need more research on chronic immune escape mechanisms	Limited clinical correlation with long-term outcomes	Scarcity of human trials; need for translational research in endemic populations

**Table 2:** *H. pylori*–helminth/parasite co-infection by type.

Country/Region	*H. pylori* Prevalence (%)	Helminth Prevalence (%)	Co-Infection Prevalence (%)	Parasite Type	Notes
Egypt	~69.4%	~51.4%	39.8% (95% CI: 27.8–51.9)	Mixed (helminths + protozoa)	High burden of both types
Ethiopia	—	—	5.9% (95% CI: 4.1–7.6)	Mixed	One of the lowest rates
Uganda	—	—	30.2% (children)	Mixed	Intermediate rates
Nigeria	89.7%	—	~7%	Mixed	High H. pylori but low co-infection
Sudan	—	—	29.3% (pooled)	Mixed	Large variability
Colombia (Tumaco and Pasto)	~93%	25–54%	21–45% (children)	Helminth-only	Mainly soil-transmitted helminths
Mexico	—	—	50–70% (in *H. pylori*-infected children)	Mixed	Multiple intestinal parasites
Iran	—	—	10–30% (in *H. pylori*-positive children)	Mixed	Protozoa + some helminths
Turkey	—	—	15–45% (symptomatic children)	Protozoa-only	Primarily *Giardia lamblia*
Yemen	~30% (dyspeptic patients)	—	—	Protozoa-only	Mainly *Entamoeba histolytica*, *Giardia*
Thailand	—	—	—	Helminth-only	*Opisthorchis viverrini*; cancer risk
Colombia (low gastric cancer areas)	—	—	—	Helminth-only	Higher *Ascaris lumbricoides* seropositivity
Global pooled	—	—	31% (95% CI: 18.66–43.39)	Mixed	High heterogeneity

## Data Availability

There is no data to support the findings of this review.
